# Recent Clinical and Experimental Advances in Atrial Fibrillation

**DOI:** 10.5402/2011/958189

**Published:** 2011-08-01

**Authors:** Shigeru Miyagawa, Taichi Sakaguchi, Hiroyuki Nishi, Yasushi Yoshikawa, Satsuki Fukushima, Shunsuke Saito, Yoshiki Sawa

**Affiliations:** Department of Cardiovascular Surgery, Osaka University Graduate School of Medicine, 2-2 Yamada-oka, Suita, Osaka 565-0871, Japan

## Abstract

Atrial fibrillation (AF) is the most common arrhythmia in clinical settings (Fuster et al., 2001), and it is often associated with congestive heart diseases (Issac et al., 2007). Many studies in both laboratory and clinical settings have sought to analyze the mechanisms of AF, develop treatments based on these mechanisms, and examine atrial remodeling in chronic AF. The aim of this paper is to analyze recent findings regarding the atrial remodeling that occurs in AF. In particular, we will describe the electrical and structural changes that involve atrial myocytes and the extracellular matrix. We will also describe the general classification and basic pathophysiology of AF and its surgical treatments.

## 1. Classification of AF

The joint American College of Cardiology/American Heart Association/European Society of Cardiology proposed a classification system for AF to simplify its heterogeneous clinical aspects and clarify its clinical states [1]. Patients are initially classified as having a “first detected episode of AF,” when AF is confirmed by clinicians. If a patient has two or more episodes, the AF is classified as recurrent. Recurrent AF is designated as paroxysmal or persistent. Paroxysmal AF is an episode that generally continues for 7 or fewer days and terminates on its own. Persistent AF usually continues for more than 7 days without self-terminating and requires clinicians to terminate it using pharmacological treatment or electrical cardioversion to restore the sinus rhythm. Permanent AF is a situation in which the sinus rhythm cannot be sustained after cardioversion, and further medical efforts are required to restore it.

## 2. Pathophysiology of AF

### 2.1. The Basic Mechanisms of AF

Many researchers agree that inflammation [2], neurohormonal disorders [3], cardiovascular diseases such as valvular diseases [4], diabetes, hypertension, congestive heart failure, myocardial infarction [5], and genetic factors [6] are “modulating factors” that can induce AF. 

Classically, AF mechanisms are described by the concept of atrial ectopic foci [7], which fire spontaneously in the atrium, a single reentry circuit, or multiple reentry circuits [8, 9]. The surgical maze procedure is designed to block the multiple reentry circuits and create an isolated electrical lesion in the atrium [10]. Haissaguerre et al. reported that triggers located in the pulmonary veins initiate most cases of paroxysmal AF [7], while in some cases the trigger, such as a venous remnant in the left atrium (LA) and superior vena cavae, occurs outside the pulmonary vein. This finding supports the idea that a pulmonary vein isolation technique can cure paroxysmal AF in most cases. 

Wyse and Gersh summarized the mechanisms of AF schematically [11]. In the basic scheme, a trigger and substrate invoke reentry in the atrium, and the firing of a focus leads directly to AF. AF itself leads to electrophysiological and structural atrial remodeling and produces modulating factors that continue to initiate AF, leading to permanent AF. Numerous factors, such as triggers, substrates, modulating factors, AF itself, and atrial remodeling greatly impact one another and the perpetuation of AF. Many experimental and clinical studies on the mechanisms of AF have led to numerous theories and insights. However, the factors that induce AF are very complicated, and AF still remains only partially understood.

### 2.2. Atrial Remodeling of Chronic AF

There are many papers on the electrophysiological and structural atrial remodeling that takes place in chronic AF patients. In particular, dramatic structural changes in the atrial myocytes and extracellular matrix (ECM) have been demonstrated along with changes in the electrical properties of myocytes. Paroxysmal AF sometimes leads to permanent AF, and the elucidation of these changes could help us understand the mechanisms by which perpetual AF is established.

### 2.3. Electrical Changes

Transmembrane ionic currents play a crucial role in the mechanisms of AF and impact the contraction of atrial muscle. Many kinds of transmembrane ionic currents have been reported, including the inward rectifier potassium current (*I*_K1_), sodium current (*I*_NA_), transient outward potassium current (*I*_to_), ultrarapid component of *I*_K_(*I*_Kur_), rapid component of *I*_K_  (*I*_Kr_), slow component of *I*_K_  (*I*_Ks_), inward L-type Ca^2+^ current (*I*_Ca_), and transient inward Na^+^/Ca^2+^ exchanger (NCX). The action potential (AP) and action potential duration (APD) in atrial myocytes depend on the balance between the inward and outward ionic currents; this balance affects the refractory period and may be useful for the prevention of AF [9].

Many studies on AF animal models and human patients describe abnormal ionic currents in AF. Li et al. found decreases in *I*_to_, *I*_Ca_, and *I*_Ks_ and increases in the NCX current, in the atrium of a dog rapid ventricular pacing model that induced heart failure [12]. On the other hand, in a dog atrial pacing model, Yue et al. demonstrated decreases in *I*_to_, *I*_Ca_ that were similar to the dog heart-failure model, but without a significant change in *I*_Ks_ [13]. Regarding *I*_NA_, Gaspo et al. reported that the *I*_NA_ density was reduced in a dog atrial pacing model, in a patch-clamp study [14]. In addition, although the effective refractory period (ERP) and APD were decreased by rapid atrial pacing [13, 15], in a ventricular rapid pacing model the APD either did not change (slow rate) or increased (faster rate) [12], and the ERP showed no change [16]. 

Thus, there are discrepancies between the atrial pacing model and ventricular pacing model in terms of ionic change and the AP of atrial myocytes. It is clear that the mechanisms are quite different between AF that is based on heart failure and that originating from the atrium. Thus, different treatments are used for AF cases of different etiology. Van Wagoner et al. reported a significant reduction in *I*_Ca_, no changes in Ca^2+^ channel performance, and a correlation between the preoperative *I*_Ca_ density and the incidence of postoperative AF, using isolated human atrial myocytes [17]. In another human study, Bosch et al. demonstrated a marked shortening of the APD, a reduction in *I*_Ca_ and *I*_to_, and an increase in *I*_K1_ [18].

We summarized the abnormalities in the ionic currents in AF in animal and human studies and found that a reduction of *I*_Ca_ and *I*_to_ is common. The relevance of these reductions to the maintenance of AF was determined by Nattel [9]. AF induces Ca^2+^ entry into the cytoplasm of atrial myocytes through Ca^2+^ cannels, leading to a high concentration of Ca^2+^ in the cell [19], which threatens cell viability. The cell protects itself from further Ca^2+^ loading by inactivating *I*_Ca_ [20] and reducing the levels of mRNA [21] and proteins [13, 17, 18] responsible for the *I*_Ca_. However, reducing the *I*_Ca_ leads to reductions in APD and ERP, resulting in the induction and maintenance of AF. This was confirmed by Ausma et al., who showed by electron microscopy that significant increases in Ca^2+^ deposits were visible at the sarcolemma, T-tubes, and mitochondria of atrial cardiomyocytes, in a goat atrial rapid pacing model [22].

### 2.4. Structural Changes

In chronic AF, the most dramatic change is the dilatation of the atrium wall by mechanical stretching, which is induced by hemodynamic overload [23, 24]. Furthermore, left atrial enlargement has been proposed to be closely related to atrial diastolic [25] and systolic dysfunction [26]. This prolonged mechanical stretching induces changes in the expression and localization of atrial myocardial proteins, in the ECM, and in electrophysiological activity, and some of these changes may be irreversible [27]. In this section, we analyze the structural changes associated with AF, as determined from animal experiments and human tissues (Table 1).

### 2.5. Changes in Cardiomyocytes in AF Patients and Animal Models

#### 2.5.1. Human Studies

Studies to determine the atrial structural changes in humans are limited, and further studies are needed to clarify the mechanisms of human AF. Cardiac muscle cells of the left atrial tissue in mitral valvular disease show hypertrophy and, particularly in fibrotic areas, structural changes such as a proliferation of Z-band material, cytoskeletal filaments, myofibrillar loss, a proliferation of elements of free and extended junctional sarcoplasmic reticulum, variations in the size and number of mitochondria, the appearance of abnormal mitochondria, dissociated intercellular junctions, the formation of spherical microparticles, and an accumulation of lysosomal degradation products [28]. These degenerative changes are reported in several papers [29, 30]. However, the probability of hypertrophy of atrial myocytes in cases of lone AF is not high.

In contrast, lymphomononuclear infiltrates with necrosis of the adjacent myocytes are often seen in the atrial myocytes of lone AF [31]. Although the atrial myocytes in AF undergo degenerative changes, the pathological changes are secondary, and it is unknown whether there is a close relationship between the degenerative changes and AF. Rabine et al. reported a relationship between degenerative changes and electrical function [30], but the primary reasons for AF remain unclear. Nevertheless, changes in myocytes, such as their stretching, may greatly impact the structural changes and calcium homeostasis [32].

#### 2.5.2. Animal Studies

Several papers describe the induction of chronic AF by rapid pacing and examine the ultrastructural changes in the atrium using a dog [15, 39–41] or goat rapid pacing model [42, 43]. In the dog experimental model, cell loss, degenerative changes including the disruption of sarcomeres, [44] loss of myofibrils, and band contraction necrosis [16] were found, accompanied by excessive fibrosis [41], enlargement of the atrium [40], increased mitochondrial size, and disruption of the sarcoplasmic reticulum [15]. Furthermore, the fast-type cardiac myosin alpha heavy chain, a structural protein, switched to the slow beta heavy chain isomer in atrial myocytes [45]. In a goat chronic AF model, Ausma et al. demonstrated significant changes in cellular substructures, such as the accumulation of glycogen, fragmentation of sarcoplasmic reticulum, homogeneous distribution of nuclear chromatin, changes in mitochondrial shape and size, loss of myofibrils, and increased cell size. 

In the goat pacing model, changes characterizing the dedifferentiation of atrial myocyte were demonstrated, including the disappearance of cardiotin, titin, and desmin at the intercalated disk, and the reexpression of *α*-smooth muscle actin, a feature of embryonic cardiomyocytes [46]. In addition, Dispersyn et al. reported that the histological changes in the chronic fibrillating atrial myocardium in the goat rapid pacing model were similar to those seen in chronic human hibernating left ventricular myocardium [47]. These findings may indicate that ischemic changes induced by tachypacing, that is, occasional microcirculatory dysfunction in the atrium, may influence structural changes in the AF atrium. 

Some degenerative changes seen in animal models are similar to those seen in human AF, but there are no reports in animal models describing the accumulation of lysosomal degradation products, which is the most typical degenerative change in the human atrium [48]. Furthermore, there are no reports of human cases showing the dedifferentiation of atrial myocytes that was demonstrated in the goat atrial fibrillation model. In human AF, there is a large quantity of inducer, such as a trigger and modulating factor, whereas in animal models the inducer is only the high pacing rate of the atrium or ventricle. Because the etiology of the animal model is quite different from that of human AF, the animal high-rate pacing models may not mimic human AF completely. However, the animal high-rate pacing model is the only one available for experimentally reproducing AF, and it has been very useful for investigating AF's pathophysiology.

In addition to elucidating the structural changes that accompany chronic AF, to know at what point AF becomes incurable, it is important to learn the time course of these changes and when they become irreversible. In a 7-hour atrial pacing dog model, atrial electrical remodeling starts within 30 minutes of very rapid pacing; this remodeling is reversible, because verapamil blocks it [53]. In a 1-week atrial pacing goat model, homogeneous chromatin distribution and gradual changes in the mitochondria and sarcoplasmic reticulum were found [43]. At this stage, electrical remodeling is reversible, but some ultrastructural changes may not be, because myocytes showed the first cellular changes, as described above. Because of the reversibility of electrical remodeling at this stage, cardioversion is effective for recovering the sinus rhythm, but AF may recur if the ultrastructure of the atrium remains abnormal. In the goat pacing model, over the long term (more than 5 weeks of pacing), the cellular and extracellular changes become so severe that they may be irreversible [16]. On the other hand, Ausma [27] reported that electrical and structural remodeling after prolonged AF (4 months) was reversible in a goat right atrium pacing model. The ERP was normal 2 months and sinus rhythm 4 months after the end of the induced AF. However, although some ultrastructural changes (atrial myocyte diameter, number of myocytes with severe myolysis and Connexin 40) returned to normal, the fibrosis and changes in some structural proteins remained abnormal, and paroxysmal AF episodes occurred after the pacing. This report indicates that the remaining ultrastructural changes have the potential to induce the recurrence of AF, despite the complete reversal of the electrical changes.

However, the time course of changes in the pathophysiology of human AF has not been demonstrated. In humans, inducers of AF include volume-pressure overload, such as heart failure, and cardiac diseases such as valvular and coronary artery diseases, not rapid atrial pacing. Therefore, there is no scientific basis for the reversibility of electrical and structural remodeling in humans that could provide information for the effective treatment of AF.

### 2.6. Changes in Intercellular Connections: Gap Junctions

In myocytes, the electrical conductivity between cells is sustained by the Connexin located in the gap junctions. This conductivity is responsible for the regular heart rhythm and synchronous contraction. Connexin is expressed in every tissue and organ and consists of multiple subtypes; 20 subtypes of Connexin have been found in mouse and 21 in human [54]. In particular, Connexins 43, 40, and 45 are expressed in the human right atrium [55, 56].

In chronic overload of the atrium in a rat myocardial infarction model, the lateral membranes of myocytes show high levels of nonphosphorylated Connexin 43 [35]. In the high-rate pacing goat model, the homogeneous Connexin 40 distribution pattern changed significantly and became heterogeneous, that is, a mixture of Connexins 40 and 43 with increasing duration of the atrial pacing. In contrast, the regions in which Connexin 43 was the sole Connexin remained unchanged [57]. A reduction in Connexin 40 in a chronic AF goat model was observed in another study [27], and this was also seen in a canine model [39]. Thus, Connexin remodeling has been seen in all animal models of chronic AF. In samples from human AF patients, several studies have reported that Connexin 40 is highly expressed on the lateral membrane of atrial cells [33, 36]. On the other hand, Kostin et al. reported the lateralization of Connexins 43 and 40 in human samples but observed that the expression of Connexin 40 and 43 decreased in the AF atrium [37]. Some studies agree with this finding for Connexin 40 expression [34]. In contrast, two others report that the expression of Connexin 43 does not change in AF patients [36].

There are many discrepancies among the studies on Connexin 40 and 43 expression and localization in human AF. These discrepancies may be owing to differences in the patients' background, for example, their age, their degree and duration of AF, whether the experiments were performed after a surgical procedure or not, and differences in the location of the tissue examined. Moreover, in animal AF models, AF is induced via many disparate routes, including by ventricular tachypacing in a congestive heart failure model, by atrial tachypacing an atrial tachycardia model, by myocardial infarction, and by hypertension, in dogs [39], goats [27, 38, 57], and rats [35]. In humans, most cases of AF are caused by mitral valve disease [58] or ischemic cardiomyopathy [59]. Therefore, the animal models are not completely relevant to human AF. Consequently, there are large discrepancies between the results from human studies and animal experiments regarding the histological and molecular changes in AF. 

Nevertheless, the mechanism of Connexin remodeling is considered a key factor in the primary mechanisms underlying AF. Further studies of Connexin 43 and 40 are needed to clarify the mechanisms of Connexin remodeling and its role in human AF.

### 2.7. Changes in Extracellular Matrix

Changes in the extracellular matrix are quite dramatic in AF patients, and they have been described in many papers. Fibrosis is a typical histological change in human AF [37, 49] and in dog and [16, 41, 60, 61] rat AF models [52]. Fibrosis is thought to be a key factor in the AF-associated remodeling of Connexins and electrical conduction [35, 62]. However, in one study using a canine model of heart failure induced by right ventricle rapid pacing, the total atrial collagen content did not change, although a disarray of collagen fibers was demonstrated [51]. The maintenance of total collagen content was attributable to the model being a heart failure model rather than an atrial pacing model, and the report also demonstrated that collagen synthesis correlated well with the left atrial wall tension index. In particular, the expressions of collagen type-1, which is the main collagen in heart [37], and collagen VI [49] were significantly different in the atrium of AF patients compared with sinus rhythm patients. On the other hand, Li et al. reported significant interstitial fibrosis in a dog model of heart failure induced by rapid ventricular pacing, compared with a rapid atrial pacing model [16]. 

Recent reports indicate that serum markers of collagen type-1 are critical parameters for distinguishing paroxysmal and persistent AF [50]. This important finding enabled AF to be diagnosed easily by a noninvasive method, serum sampling. However, because the serum levels of these collagen markers may also be elevated in liver fibrosis, systemic sclerosis, and other collagen diseases [63, 64], it is necessary to check not only the serum markers but also histological changes to diagnose the AF stage. 

In general, Matrix metalloproteinases (MMPs) can degrade almost all ECM proteins and thereby control tissue remodeling in both normal and diseased organs [65–67]. Tissue inhibitor of matrix metalloproteinases (TIMPs) can inhibit the activity of MMPs [68]. A typical histological change in the AF atrium is fibrosis, as mentioned above, and its occurrence may depend on the balance between the expression of MMPs and TIMPs, as it is in other organs. Several reports have demonstrated that a disturbed MMP/TIMP system is a major reason for the fibrosis observed in left ventricular heart failure [69]. 

Several papers have described changes in MMPs in AF. MMP-9 and MMP-2 are overexpressed in the atrium in a canine model induced by right ventricle rapid pacing [51]. On the other hand, in a rat myocardial infarction model, MMP-7 was overexpressed in the atrium [52]. In one human study, Polyakova et al. reported that MMP-2 and -9 were significantly increased in AF patients compared with sinus rhythm patients [49], but in another, no difference in MMP-9 was found between these patient groups [70]. Clearly, there are great discrepancies in the MMP expression patterns reported, and comparisons among these studies are not meaningful, because of the different species, different background diseases, and stages of AF studied. Thus, experiments using similar conditions, including the same experimental species, background diseases, and AF stage, are needed to clarify the changes in MMPs. Nevertheless, it is safe to say that MMPs show significant changes in AF that coincide with dramatic structural changes in the atrium. 

Regrettably, the situation is similar for studies of TIMP changes. In a human study, serum TIMP-1 showed significant changes [50], but another paper reported no change in TIMP-1 when human tissue samples were analyzed [70]. Another paper showed significant AF-associated overexpression of TIMP-1 but no significant change in TIMP-3 or -4 [49]. Nevertheless, we speculate that the MMP/TIMP system in the AF atrium is damaged, and this damaged system leads to significant atrial fibrosis (Table 2).

Other factors that can regulate ECM remodeling include reversion-inducing cysteine-rich protein with Kazal motifs (RECK) [71] and transforming growth factor (TGF-*β*1) [72]. Polyakova et al. reported that the expression of RECK, an alternative MMP inhibitor, increased and that the levels of both TGF-*β*1 and Smads were high in AF patients compared with sinus rhythm patients. They concluded that RECK and the TGF-*β*1-Smads pathway are important for ECM remodeling in the AF atrium [49]. In addition, in a dog ventricular tachypacing model, TGF-*β*1 increased in the atrium [61].

Another profibrotic molecule, angiotensin II (AT-II), is well known to be involved in organ fibrotic changes, such as hypertensive heart disease [73], chronic heart failure [74], myocardial infarction [75], and cardiomyopathy [76]. In a dog ventricular tachypacing model, the tissue atrial AT-II levels increased, and an ACE inhibitor could prevent this increase [60]. Another paper showed that the tissue concentration of AT-II in the atrium increased, reaching a maximum level at 24 h, and that this increase was slow compared with the increase in the left ventricle, in a dog ventricular tachypacing model [61]. AT-II promotes interstitial fibrosis and plays crucial roles in atrial remodeling in AF. Moreover, AT-II can activate the TGF-*β*1-Smads pathway, which promotes the expression of collagen type I and collagen type II [77].

The phenomenon of fibrosis in the atrium is secondary to AF, and the MMP/TIMP system, RECK, and the TGF-*β*1-Smads pathway may be the primary effectors of the fibrosis. These mechanisms affect each other, adding complexity to the situation. Moreover, other primary mechanisms might also be involved in fibrosis. Further studies are needed to clarify the mechanisms of fibrosis and ECM remodeling in AF.

### 2.8. Apoptosis

Apoptosis plays a key role in the mechanism of organ failure in several different organs [78] and in AF. In a sheep elevated blood pressure model, apoptosis increased in the atrium [79], and in a pig atrial tachypacing model, apoptotic changes were found in the atria [80]. Cardin et al. demonstrated in a canine ventricular tachypacing model that apoptosis, with an elevated ratio of the proapoptotic protein Bax to the antiapoptotic protein Bcl-2, increased after 24 hours of ventricular tachypacing; this change was maintained for one week and then declined to baseline [81]. This study also showed that the apoptotic changes led to interstitial fibrosis that peaked at 5 weeks [81]. Therefore, a large quantity of atrial myocytes may be removed by apoptosis in the initial phase of AF and replaced, subsequently, by fibroblasts, which express collagen type-1. 

In a goat atrial pacing model that extended 9–23 weeks, no apoptotic changes were observed in the atrium, based on the levels of Bcl-2, P53, and PCNA, although the atrium showed fibrotic changes [47]. This paper supports the above-mentioned hypothesis that fibrosis results from the balance of MMPs and TIMPs. On the other hand, in samples of patients who had suffered from AF for at least 1 month, a high percentage of abnormal myocytes was found; most were TUNEL-positive cells that showed increased caspase-3 expression, decreased BCL-2 expression, and unchanged Bax expression [82]. In spite of some discrepancies between humans and animal models, both lines of evidence indicate that apoptosis may play a large role in the structural remodeling of AF.

## 3. Atrial Fibrillation after Cardiac Surgery

AF is a common complication after cardiovascular surgery [83]. The incidence of AF after heart surgery is 10% to 65% and is associated with an increased the risk of stroke [84]. Postoperative AF prolongs the duration of the hospital stay after surgery [85], so its prevention and treatment are important for shortening the time a patient must remain in the hospital. Furthermore, the etiology of AF after an operation is apparently quite different from that of AF in non-post-operative patients. It is multifactorial, and it is not understood clearly, so its prevention and treatment are still unsatisfactory. In this section, we will summarize what is known about the mechanisms of AF after cardiac surgery. 

Several possible pre-operative and postoperative mechanisms have been proposed to explain postoperative AF. In the pre-operative stage, advanced age [86, 87], genetic predisposition [88], and the nonuse of a beta-adrenergic blocker [86, 87] have all been shown to correlate with an increased incidence of postoperative AF. At the molecular level, Gaudino et al. demonstrated that the likelihood of postoperative AF is increased by a 174 G/C interleukin-6 promoter gene variant and high serum levels of interleukin-6 and fibrinogen and that the GG genotype is an independent predictor [88]. 

In addition, in the postoperative stage, inflammation induced by heart surgery plays an important role in initiating AF. In a canine model, Tselentakis reported that acute inflammation slows conduction in the atrium anisotropically and induces reentry [89]. Moreover, invasive surgery, such as pericardiotomy or atriotomy, induces inflammation and leads to inhomogeneous atrial conduction and prolongation of AF in a canine model; this phenomenon is blocked by an anti-inflammatory drug [90]. Bruins et al. reported that interleukin-6, C-reactive protein (CRP), and complement-CRP complex, respectively, reached their maximum levels at 6 hours, two days, and two to three days after surgery [91]. Furthermore, an elevated white blood cell count, a common marker of inflammation, is an independent predictor of postoperative AF [87, 92]. In addition to these parameters, other mechanisms that may contribute to the incidence of postoperative AF include atrial oxidative stress [93], postoperative complications [86, 87], and impaired renal function following heart surgery [94]. 

By contrast with the other mechanisms mentioned, Connexin appears to play a role in postoperative AF as well as other etiologies. One paper showed a significant increase in the expression of Connexin 40 in patients who suffered from AF after coronary artery bypass grafting [59]. Similarly, Dupont et al. reported that Connexin 40 mRNA and protein were expressed at high levels after coronary artery bypass grafting and that the protein had a heterogeneous distribution, although neither Connexin 43 nor 45 changed [36]. Another paper demonstrated that the postoperative expression levels of Connexin 40 and 43 differed considerably for surgeries involving cardioplegic arrest or a beating-heart technique, and this difference might affect the incidence of postoperative AF [95]. Cardiovascular surgeons should be aware that open-heart surgeries bring about molecular changes that can lead to complications such as postoperative arrhythmias and that the degree of molecular change depends on the method of the operation.

## 4. The Mechanisms of “AF Begets AF”

We have summarized and discussed the histological and electrical events occurring in chronic AF. Another important aspect, in terms of preventing permanent AF, involves elucidating the mechanisms that lead from paroxysmal AF to permanent AF. The mechanisms underlying this change are likely to be very complicated, but several review articles have presented hypotheses to account for them [9, 11, 96]. 

**Figure 1:**
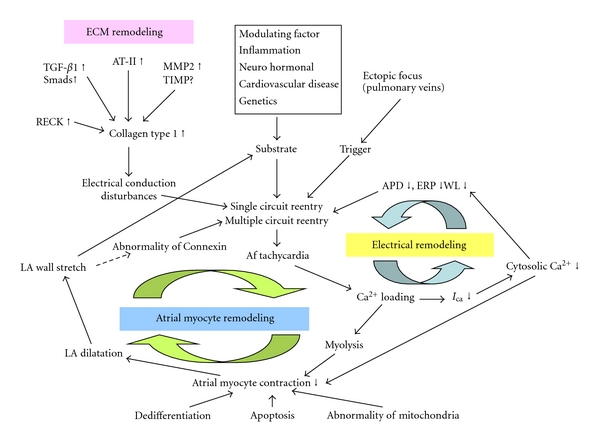
Mechanism of “AF begets AF.”

We have summarized the mechanisms for the progression of paroxysmal or persistent AF in Figure 1. Once AF is initiated by a trigger or substrate, the tachycardia induces electrical remodeling. This becomes a vicious circle that leads to permanent changes. In addition, Ca^2+^ loading, which is induced by tachycardia, attenuates contraction of the atrial myocytes, resulting in left atrial (LA) dilatation, which can be a type of substrate in AF. 

The structural remodeling of atrial myocytes involves dramatic histological changes, such as abnormal mitochondria and the dedifferentiation of atrial myocytes, and these changes can also affect myocyte contraction. In ECM remodeling, collagen type-1 synthesis is upregulated by AT-II, the MMP/TIMP system, RECK, and the TGF-*β*1-Smads pathway. The increase in collagen type-1 induces LA dilatation and disturbances in the electrical conduction between atrial myocytes. As described above, there is a complicated network among electrical remodeling, atrial myocyte structural remodeling, and ECM remodeling. Once AF is initiated by some trigger or substrate, it may become permanent stage via the collective and intertwined effects of these complicated mechanisms. This process is called “AF begets AF.”

## 5. Future Prospects

### 5.1. ECM Cellular Connection

Various studies have demonstrated that the content of the ECM, fibrosis, the MMP/TIMP system, and other factors promote interstitial fibrosis. However, there are no reports on the ECM cellular connection in AF. Another key to the mechanisms of AF may be cell-matrix communication, in which integrin is the most important molecule for cell-matrix adhesion. In the absence of cell-matrix interactions through integrin, cells of every type, except blood cells, fall into a type of apoptosis called “anoikis.” Cells also exert various functions through integrin-mediated cell-matrix interactions, including proliferation, differentiation, motility, and protein production. In AF patients, the connection between cells and the ECM might be disturbed, leading to abnormal contraction and distressed electrical conductance in the atrium. Moreover, nobody knows which integrin subtypes exist on the membrane of atrial myocytes, although the integrin subtypes in ventricular myocytes have been well elucidated. In addition, the roles of Laminins, which are integrin antagonists, are not fully understood in AF. As with the ECM cellular communication, there are no reports on the intercellular signaling pathways involving integrins in AF. Studies on the roles of ECM cellular communication and the intercellular signal pathways in AF may elucidate the primary mechanisms of structural remodeling in chronic AF. It is important to determine which changes initiate atrial remodeling, which ones have secondary effects.

### 5.2. Establishment of Clinical Criteria to Determine Whether AF Is Reversible

In clinical settings, decisions about the severity of AF are determined by the result of treatments, such as the effectiveness of cardioversion, and the duration of AF. It is important to establish a set of criteria, such as histological, molecular, and electrical parameters, by which clinicians can decide whether AF is reversible or not. In addition, developing criteria that could allow noninvasive methods, such as MRI, to be used for diagnosing the severity of AF would be extremely beneficial. If we can elucidate criteria to determine when AF is curable by medication or ablation, it will greatly advance the prevention and treatment of AF.

## 6. Conclusion

Basic researchers have performed many excellent studies on AF, but controversies remain. To resolve these controversies, researchers need to analyze the complicated mechanisms of AF through carefully controlled, systematic experiments. 

Clinicians have valiantly sought to treat AF by developing surgical methods, ablation-based catheter techniques, and pharmaceuticals. However, the prevention and treatment of AF remain unsatisfactory. The results of basic research that will allow clinicians to determine whether patients have reversible or irreversible AF and which treatment is appropriate for a given patient are urgently needed. Furthermore, such studies may lead to new strategies for preventing or curing AF completely.

## Figures and Tables

**Table 1:** Alteration in Connexin expression in AF.

Author	Species	Disease animal model	Sample	Cx protein	Cx mRNA	Histology	Others
Luo	Human *n* = 24	AF	RA appendage	Cx43 ↓ (volume fraction)			
Polontchouk et al.	Human *n* = 12	Chronic AF	Atrial tissue	Cx43→, Cx40 ↑ (western)		Lateral membrane of atrial cells	
Nao et al.	Human *n* = 10	Chronic AF	RA myocardium	Cx40 ↓,Cx43→ (western)	Cx40 ↓ Cx43→(PCR)		Cx40 abnormal phosphorylation
Rucker-Martin et al.	Human rat	Chronic AF MI	RA appendage	nonphosphorylated Cx43 ↑			Myocyte-myocyte coupling ↓
Dupont et al.	Human *n* = 9	CAD with AF	RA appendage	Cx43,45→ Cx40 ↑ (western)	Cx43,45→ Cx40 ↑ (northern)		
Li	Human *n* = 45	CAD with AF	RA appendage	Cx40,43→ (western)			
Kostin et al.	Human *n* = 31	Chronic AF	RA free wall appendage	Cx 40,43 ↓ (immunoconfocal)		Lateralization of Cx40,43 Cx40 heterogenous distribution	
Dhein	Human *n* = 126	Chronic AF	LA free wall		Cx40,43 ↑ (PCR)		Cx40 expression parallel to serum Ca
van der Velden et al.	Goat	RA pacing	RA, LA appendage	Cx40,43→ (western)	C × 40,43→ (PCR)	Cx40 inhomogeneities No change of Cx43 distribution	
Ausma et al.	Goat	RA pacing	Atrial appendage	Cx40↓ (western)			
Zhang et al.	Dog	RA pacing	PV, LA tissue	PV; Cx40 ↓ LA;Cx43↓ (western)	PV,LA;Cx40 → Cx43→(PCR)		
Van der Velden	Goat	RA pacing	RA, LA appendage	Cx40/Cx43↓ (immunofluorescence signal)	Cx40,43 → (PCR)	Heterogeneity in Cx40	
Elvan	Dog	RA pacing	RA tissue	Cx43 ↑ (immunohistochemical analysis)			

CAD: coronary artery disease; RA: right atrium; LA: left atrium; Cx: Connexin.

**Table 2:** Alteration in MMP and TIMP expression in AF.

Author	Species	Diseases animal model	Sample	MMP protein	MMP mRNA	MMP activity	TIMP protein	TIMP mRNA
Nakano	Human *n* = 25	Paroxysmal AF Chronic AF	RA appendages	MMP9 ↑ (ELISA)	MMP9 ↑ (PCR)	Active MMP9 ↑ Latent MMP9 ↑ Active MMP1→ Latent MMP1→ Active MMP2→ Latent MMP2→ (western)	TIMP1→ (ELISA)	TIMP1→ (PCR)
Climent	Human *n* = 46	Persistent AF	Blood	MMP1 ↓ (ELISA)				
Gramley	Human *n* = 146	Paroxysmal AF Persistent AF	RA appendage	MMP2,9→	MMP2,9→ (PCR)	MMP2,9↑ (zymography)		TIMP1↓ TIMP2↓
Mukherjee	human *n* = 23	Chronic AF	RA,LA wall	RA MMP1,8↑14↓		LA MMP9 ↑ (zymography)	RA TIMP1,2,3,4→ LATIMP3↑ (Western)	
Xu	Human *n* = 53	DCM, End stage HF with AF	Atrial myocardium	MMP2,9↑ (western)		MMP2,9↑ (zymography)	TIMP1→ TIMP2↓ (western)	
Martin	Human *n* = 48	Chronic AF	Blood	MMP1 ↓ (ELISA)				
Polyakova et al.	Human *n* = 24	Chronic AF	RA appendages RA free walls	MMP2,9↑ (western)			TIMP1,2↑ TIMP3,4→ (Western)	
Kallergis et al.	Human n=70	paroxysmal AF persistent AF	Blood	MMP1↑in paroxysmal versus persistent (ELISA)			TIMP1↑ (versus control)	
Anne	Human *n* = 9	Permanent AF	RA, LA appendage	MMP1↓ (western) (MVS with SR and AF)		MMP9↓ (zymography) (MVS with SR and AF)		
Khan et al.	Canine	RV pacing	Atrial tissue			MMP2,9↑ (zymography)		
Chen	Pig	RA pacing	Atrial tissue	MMP9↑ MMP2→	MMP9↑ MMP2→ (PCR)		TIMP1,3↑ TIMP2→	
Boixel et al.	Rat	MI	LA myocardium	MMP2,7,13↑ (western)		MMP2,7↑ (zymography)	TIMP1,2,4→ (western)	

MI: myocardial infarction; SR: sinus rhythm; MVS: mitral valve surgery.
